# Improving the abrasion resistance of Ti6Al4V alloy by modifying its surface with a diazonium salt and attaching of polyurethane

**DOI:** 10.1038/s41598-020-76360-3

**Published:** 2020-11-06

**Authors:** Mariusz Sandomierski, Tomasz Buchwald, Adam Patalas, Adam Voelkel

**Affiliations:** 1 Institute of Chemical Technology and Engineering, Poznań University of Technology, Berdychowo 4, 60-965 Poznan, Poland; 2 Institute of Materials Research and Quantum Engineering, Poznań University of Technology, Piotrowo 3, 60-965 Poznan, Poland; 3 Institute of Mechanical Technology, Poznań University of Technology, Piotrowo 3, 60-965 Poznan, Poland

**Keywords:** Chemistry, Materials science

## Abstract

Commonly used endoprostheses in the orthopedic industry are those made of Ti6Al4V titanium alloy. Unfortunately, this material has low abrasion resistance, and therefore methods of their modification are still sought. A sensible approach is coating the alloy with a layer of a polymer having higher abrasion resistance. The adhesion of polymers to alloy is low, therefore the alloy requires prior modification. In this work, the alloy was modified with three types of diazonium salt and the influence of substituent on the effectiveness of modification was determined. Then, five or ten polyurethane layers were attached to the surface of the modified alloy. Using Raman mapping, the uniform distribution of layers was proved. Layers are stable in simulated human body fluids. The effectiveness of attaching subsequent layers of polyurethane was also confirmed by nanoindentation. The main focus of this work was to improve the wear resistance of the titanium alloy. The obtained results indicate that the titanium alloy with a polyurethane layer has almost ten times lower coefficient of friction compared to pure alloy. Such a low value has not been described in the literature so far. These results are the first step for obtaining endoprostheses with very high abrasion resistance.

## Introduction

Materials that play one of the most important roles in the modern world are biomaterials. Biomaterials are mainly used in orthopedics, dental care, drug delivery and tissue engineering. Biomaterials are divided into four main groups: metals and their alloys, polymers, ceramics and natural materials. In the case of biomaterials for use in orthopedics, the most important materials are titanium and titanium based alloys^1,2^. The advantage of these materials over others results from theirs biocompatibility, fatigue strength, low modulus and low density^1,3^. Ti6Al4V is the most commonly used alloy from this group as the main component of the endoprosthesis. Such a wide application results from its excellent biocompatibility and low modulus of elasticity comparable to human bone^4,5^.

Despite so many advantages, this alloy has low abrasion resistance, so many research teams are looking for a replacement for it^6^. Low abrasion resistance is particularly noticeable in the femoral and acetabular components. Due to the low abrasion resistance, the implants must be replaced after some time. In addition, it affects the release of dangerous ions causing the long-term health problems^7,8^. Till today, many different attempts have been made to modify titanium implants to improve their abrasion resistance^9^. The most commonly used methods were: thermal treatment, ion implantation, physical vapour deposition coatings, nitriding, carburization and boriding^1,10–13^. The methods improved the abrasion resistance, however, it was always associated with the deterioration of other properties^14^. Due to this, more effective method allowing the improvement of the Ti6Al4V endoprosthesis abrasion properties should be further sought.

Compared to previously presented modification methods, few research teams have so far focused on the production of thin polymer layers on the surface of titanium alloys^15^. Thin polymer coatings can allow for obtaining higher abrasion resistance of endoprosthesis surfaces and extend their use. Ghosh et al.^16^ publication is one of the few examples of works that use a polymer layer to reduce the coefficient of friction. In this work, the surface was modified using 2-Methacryloyloxyethyl phosphorylcholine polymer. Unfortunately, in this work, tribological tests were not carried out due to which there was no clear answer as to whether the goal was achieved. The second example is Liu et al. work where the surface was modified using dopamine methacrylamide—2-Methacryloyloxyethyl phosphorylcholine copolymer^17^. The results obtained in this study indicate a more than two-fold decrease in the coefficient of friction after modification. A limited number of research on this subject results from the fact that the adhesion between the polymers and the titanium surface is not satisfactory^18^. Due to this it is necessary to modify the alloy beforehand, e.g. using silane coupling agents^19^. Silane coupling agents improve the adhesion between the alloy and the polymer, however, they cannot be used in the production of any type of layer. If it is necessary to form for example a layer with hydroxyl groups, these compounds cannot be used due to the condensation reaction during the modification.

The second popular type of surface modification is the use of diazonium salts^20^. These compounds have already been used repeatedly in surface modification of different materials to obtain an aryl layer that can form covalent bonds with polymers. This type of modification was used to modify the surface of titanium materials several times and these modifications allowed to obtain poly(hydroxyethyl) methacrylate, poly(methylmethacrylate) and polyetheretherketone layers^21–23^. The results indicated that the bond strength between the polymer and titanium in the presence of the diazonium layer was much higher than for unmodified titanium. The last examples of this type of research are two works of our team^24,25^. In these works, proved the possibility of modifying the alloy with 4-hydroxymethylbenzenediazonium salt as well as the effectiveness of polyurethane attachment to the aryl layer. The obtained layers were evenly distributed over the entire alloy surface. Polyurethane was chosen as the layer covering the alloy because it has high abrasion resistance and is biocompatible as evidenced by its wide use in medicine^26,27^. Polyurethane layers have previously been obtained on a titanium surface by other research teams e.g. using spin coating technique^28^. The advantage of earlier modification with a diazonium salt over other polymer layer production techniques is that covalent bonds are formed, which results in high adhesion and greater strength of the layer.

**Figure 1:**
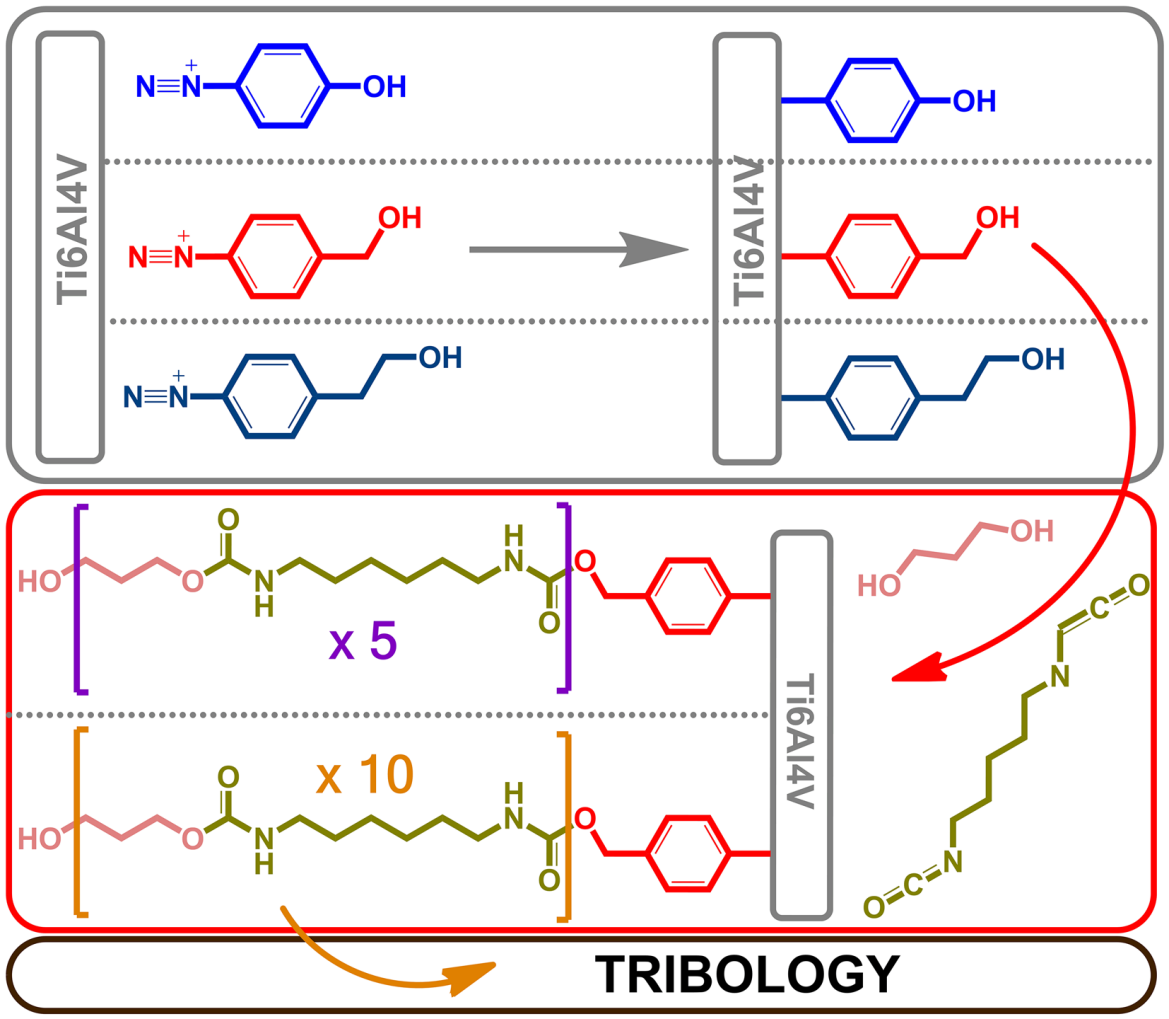
Research scheme.

In this work, the influence of the polyurethane layer covalently attached to the modified titanium alloy on its mechanical properties was determined. The research scheme is presented in Fig. 1. In the first stage, the effect of the type of substituent in the diazonium salt on the effectiveness of the modification was determined. The stability of the polymer layer in simulated body fluids was also investigated. In addition, the effect of polyurethane layers thickness on the alloy mechanical properties and their distribution on the alloy surface was determined in detail. The layers have been broadly characterized using techniques such as: Fourier-transform infrared spectroscopy, Raman spectroscopy, nanoindentation and tribology analysis.

## Materials and methods

Titanium alloy-Ti6Al4V was supplied by Arcam AB, Sweden. 4-aminophenol (≥ 98%), 4-aminobenzyl alcohol (≥ 98%), 4-aminophenethyl alcohol (≥ 98%), sodium nitrite (≥ 99%), hydrochloric acid (37%), hexamethylene diisocyanate (≥ 98%), 1,3-propanediol (≥ 98%) and reagents for the preparation of simulated body fluid were purchased from Sigma-Aldrich.

### Modification of titanium alloy surface by diazonium salt

Aromatic amine (0.44 g of 4-aminophenol or 0.5 g of 4-aminobenzyl alcohol or 0.56 g of 4-aminophenethyl alcohol) was dissolved in distilled water and cooled to a temperature below 5 °C. Then hydrochloric acid (0.4 ml) was added to the mixture. Sodium nitrite (320 mg) dissolved in distilled water was added dropwise after 30 min. Finally, Ti6Al4V plate was added to the flask after 30 min and the solution was heated to 50 °C and stirred for 2 h. The plates were then washed with distilled water and dried in 100 °C for 24 h.

Three titanium alloys modified with diazonium salts were prepared:

- Titanium alloy modified with 4-hydroxybenzenediazonium salt (Ti6Al4V–OH);
- Titanium alloy modified with 4-hydroxymethyl benzenediazonium salt (Ti6Al4V–CH_2_OH);
- Titanium alloy modified with 4-hydroxyethyl benzenediazonium salt (Ti6Al4V–CH_2_CH_2_OH).

### Polyurethane attachment

In the first step, modified plates were placed in 20 ml of hexamethylene diisocyanate for 24 h. The next step was to place the plate in 20 ml of 1,3-propanediol for 24 h. Both steps were done five (− 5L) or ten (− 10L) times. The last step was washing the surface with distilled water and drying in 100 °C for 24 h.

### Evaluation of the polyurethane layer stability in the simulated body fluid (SBF)

The SBF was prepared by dissolving the reagents in the water in the sequence listed in Table 1. The fluid was adjusted to a final pH of 7.40 at 36.5 °C by titrating aqueous 1.0 M of HCl into the SBF. The stability of the polyurethane layers on Ti6Al4V alloy surface was tested using a SBF. Samples were placed in 40 ml of the solution at 36.6 °C for one month.

**Table 1:** Reagents, their purities and amounts for preparing 1000 mL of the SBFs.

Reagents	Purity (%)	Amount
NaCl	> 99.0	8.036 g
NaHCO_3_	> 99.0	0.352 g
KCl	> 98.0	0.225 g
K_2_HPO_4_ * 3H_2_O	> 99.0	0.230 g
MgCl_2_* 6H_2_O	> 99.0	0.311 g
1.0 M-HCl	–	40 mL
CaCl_2_	> 96.0	0.293 g
Na_2_SO_4_	> 99.0	0.072 g
TRIS	> 99.8	6.063 g
1.0 M-HCl	–	0.2 mL

### Fourier-transform infrared spectroscopy (FTIR)

FTIR analysis was carried out using Vertex70 spectrometer, Bruker Optics. Materials were studied by using single reflection, diamond ATR crystal. The tests were performed at a resolution of 0.5 cm^−1^ in the wavenumber range 4000–600 cm^−1^.

### Raman spectroscopy

Raman spectroscopy analysis was carried out using in via microspectroscope, Renishaw. Materials were studied by using laser emitting 514.5 nm wavelength and Leica microscope with 20 × lens ensuring spatial resolution about 2 µm. Materials modification was confirmed by using Raman band intensity as well as background intensity. Background intensity was related with fluorescence emission of aryl and polymer layers. Laser power was constantly controlled during measurements. In this way, Raman spectra were recorded at the same conditions, and the values of band and background intensity could be directly compared between samples. The compounds distribution on alloys was determined by using Raman maps collected on 200 µm × 200 µm area with step size of 5 µm.

### Mechanical tests

Nanoindentation was carried out using a Fischer Picodentor HM1500 nanointender. Tree intents were performed on the surface of each specimens. For all indents performed, the maximum load was 300 mN (load control), holding time at maximum stress was 4 s, loading and unloading rate was 15 mN/s. In order to guarantee the accuracy of the test results, the errors of the values were calculated according to the statistical mathematics method and ISO 14577-1 standards^29–31^.

In addition to the nanoindentation tests, dry sliding wear testing was performed at room temperature using a pin-on-flat tribometer testing machine according to the ASTM G133 standard. Tribological test was carried out using a UMT-1 Brucker tribometer. The titanium samples with coating (15 mm diameter × 5 mm height) were rubbed against a hardened bearing steel ball (diameter 3/8 in.) under a constant load of 1 N with a sliding velocity of 1.25 mm/s and a total count of cycles 250 times. The friction coefficient (COF) and its change over time were measured.

## Results and discussion

**Figure 2:**
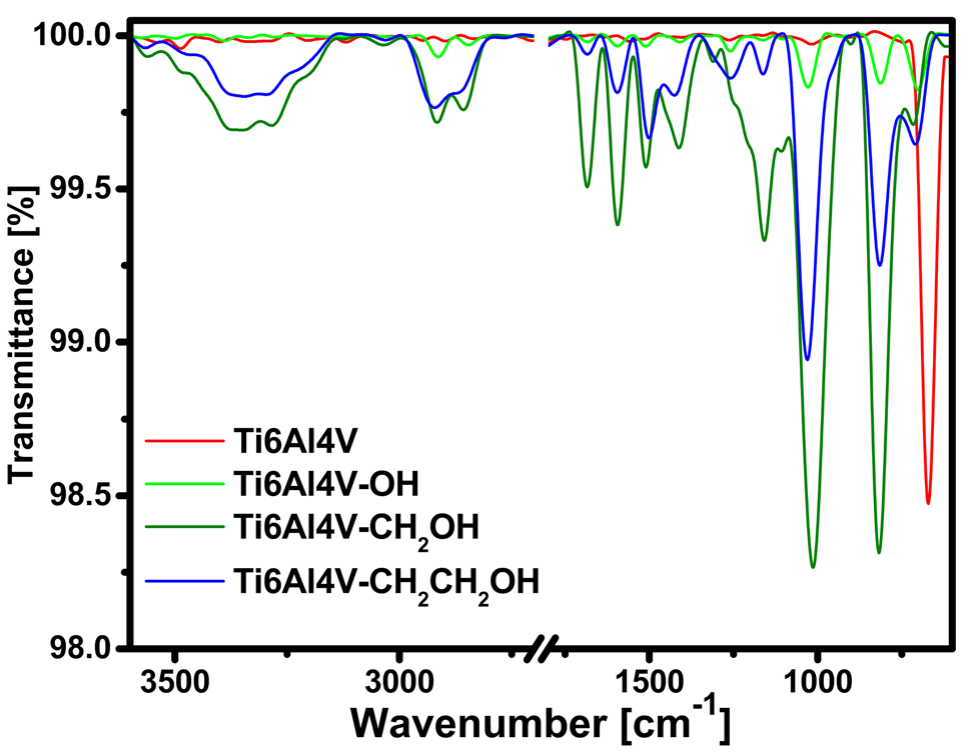
FTIR spectra of alloy before and after modification with diazonium salts.

The effectiveness of the modification using three types of diazonium salts has been determined by FTIR and Raman spectroscopy. Based on the FTIR analysis, it can be seen that the type of substituent affects the efficiency of the modification. The modification was most effective with 4-hydroxymethylbenzenediazonium salt and least effective with 4-hydroxybenzenediazonium salt (Fig. 2). The most important bands demonstrating the effectiveness of the aryl layer formation are those at ~ 1600 cm^−1^ and ~ 810 cm^−1^. These bands indicate the presence of C=C bonds in the aromatic rings present in the aryl layer. Additionally, an increase in the amount of hydroxyl groups (3250 cm^−1^) was noted on the surface of the modified alloys^32^. This is very important because these groups are to initiate the polyurethane polymerization reaction in the next step. Moreover, an increase in the number of CH_2_ groups was also noticed as evidenced by the bands at 2922 cm^−1^, 2858 cm^−1^ and 1010 cm^−1^^33^. The intensity of all bands is greatest in Ti6Al4V–CH_2_OH.

**Figure 3:**
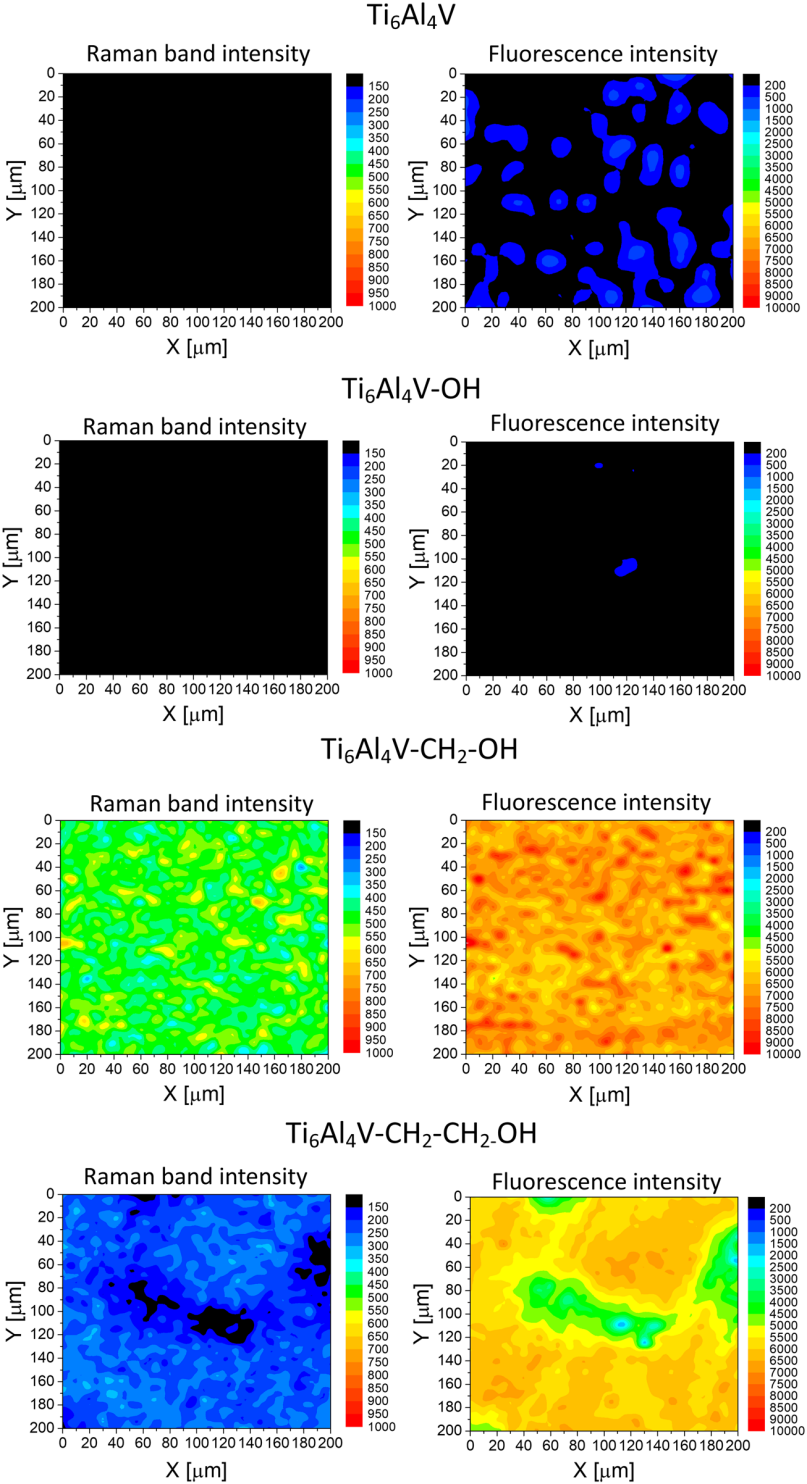
The images based on the Raman band intensity at 1600 cm^−1^ and fluorescence (background) intensity recorded on the surface of modified and unmodified alloys.

The layer obtained on the surface of the alloy should be evenly distributed. This is due to the fact that when preparing the protective layer, it should affect the properties of the final material to the same extent in all places. In the presented studies, the distribution of the layer was determined using Raman mapping (Fig. 3). The mapping was performed for fluorescence intensity (visible as background intensity in Raman spectra) and Raman band intensity at 1600 cm^−1^. This band was chosen because it is the most characteristic band occurring in the aryl layer and have been described in earlier work^24^. The mapping results are consistent with those obtained from FTIR analysis. Ti6Al4V–CH_2_OH shows the highest value of the band intensity as well as background intensity. It means that this alloy is most effectively modified. Moreover, the Raman analysis presented that Ti6Al4V–CH_2_OH surface is evenly covered. As it was already noticeable in the case of FTIR analysis, the organic layer after modification of titanium alloy with 4-hydroxybenzenediazonium salt (Ti6Al4V–OH) layer was not detected.

**Figure 4:**
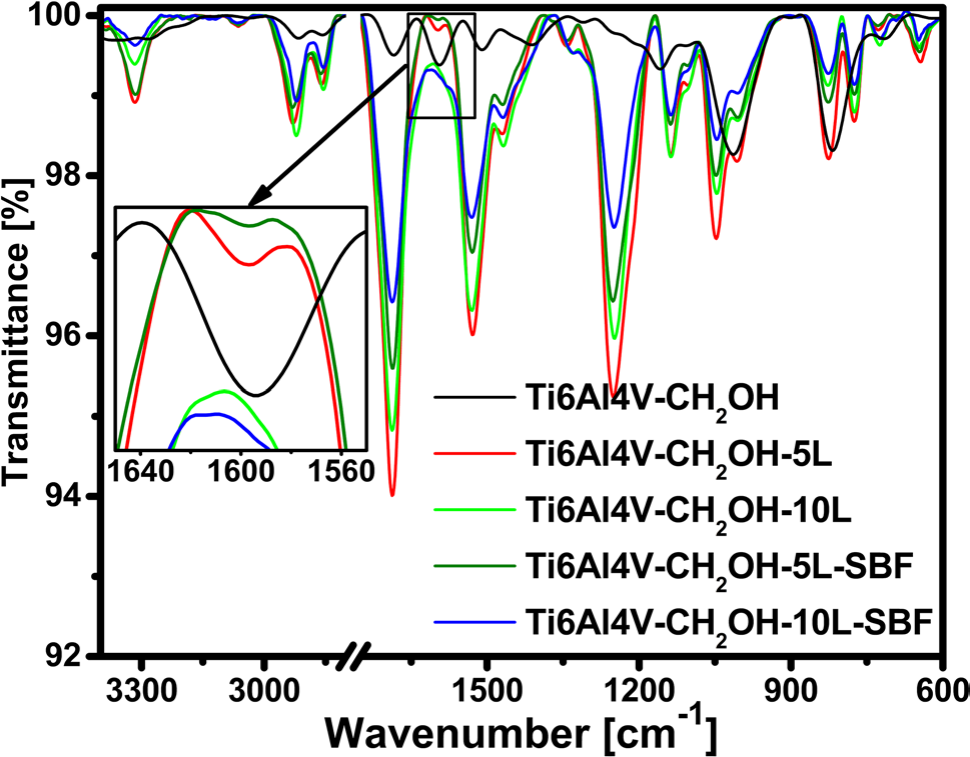
FTIR spectra of investigated materials with polyurethane layers and after placing in simulated body fluid for one month.

According to the above in the next stages of work, the difference between a titanium alloy with five and ten layers of polyurethane was determined only for titanium modified with 4-hydroxymethylbenzenediazonium salt (Fig. 4). In addition, the stability of these layers in simulated body fluids was determined (Fig. 4). After polyurethane attachment, there is a significant increase in the intensity of bands compared to a salt-modified alloy. The most important information confirming the formation of polyurethane and urethane linkages are the bands at ~ 1690 cm^−1^ and at ~ 1530 cm^−1^ assigned to C=O stretching and N–H bending vibrations, respectively^34^. The bands at 1050 cm^−1^ and 1250 cm^−1^ can be attributed to C–O bonds in the polyurethane layer. Based on the intensity of the bands, we cannot determine the effectiveness of attaching subsequent layers. However, it is visible by analyzing the FTIR results very carefully. In the case of 5 layers, the band at ~ 1600 cm^−1^ is visible which is attributed to the aromatic rings in the aryl layer whereas for 10 layers this band disappears. This confirms that the polyurethane layer in the Ti6Al4V–CH_2_OH–10L is thicker. A very important information based on the presented results is that after placing the materials in a simulated body fluid for a period of one month, no significant differences were found.

**Figure 5:**
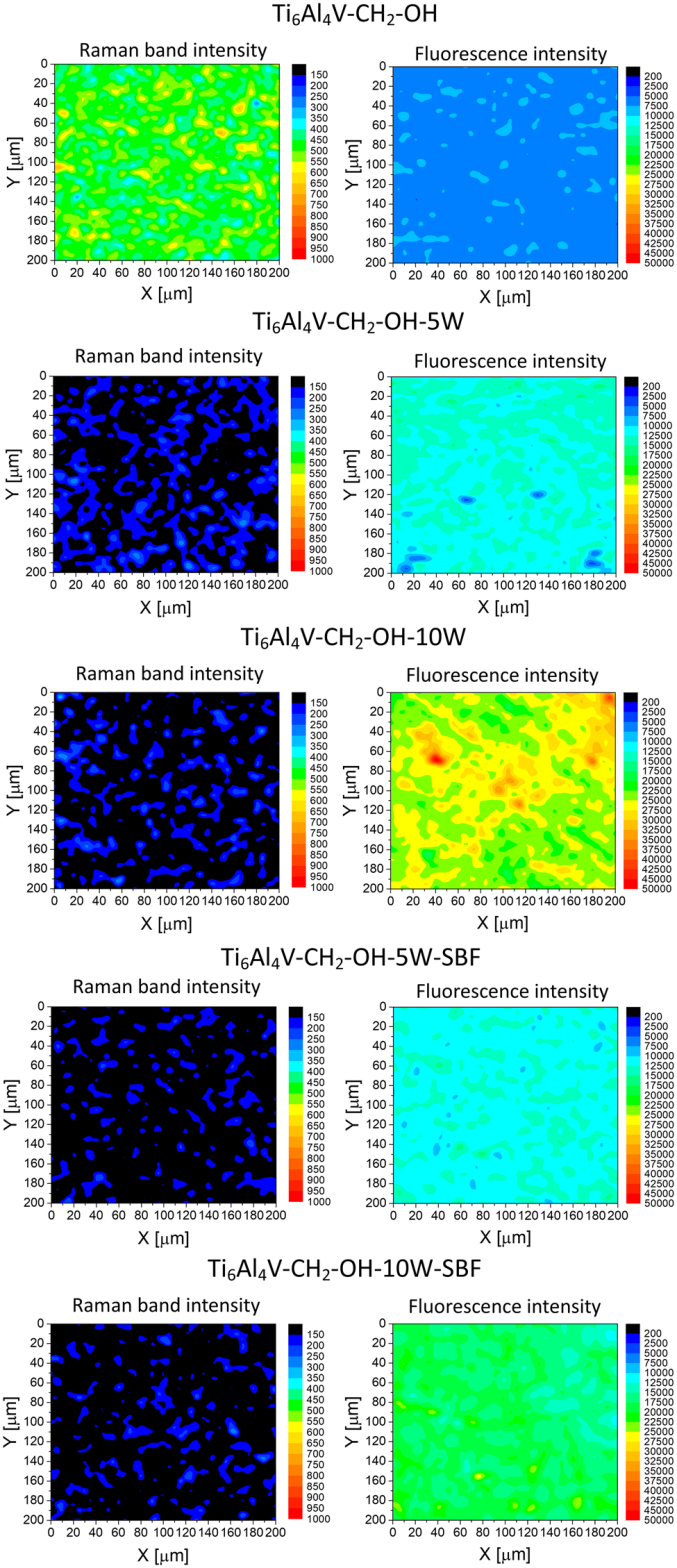
The images based on the Raman band intensity at 1600 cm^−1^ and fluorescence (background) intensity recorded on the surface of diazonium-modified alloy, alloys with polyurethane layers and alloys with polyurethane layers after placing in simulated body fluid for one month.

Raman mapping confirmed the uniform distribution of polyurethane layers on the entire surface of the titanium alloys (Fig. 5). Attaching the polymer causes the disappearance of the aromatic band, which proves that the aryl layer is covered. On the other hand, the fluorescence intensity increases significantly compared to the material modified only with a diazonium salt which indicates the formation of polyurethane. The results also show the effectiveness of attaching more polyurethane layers because the intensity for 10 layers is higher. Using this analysis, the stability of the obtained layers in the simulated body fluid was also confirmed.

**Figure 6:**
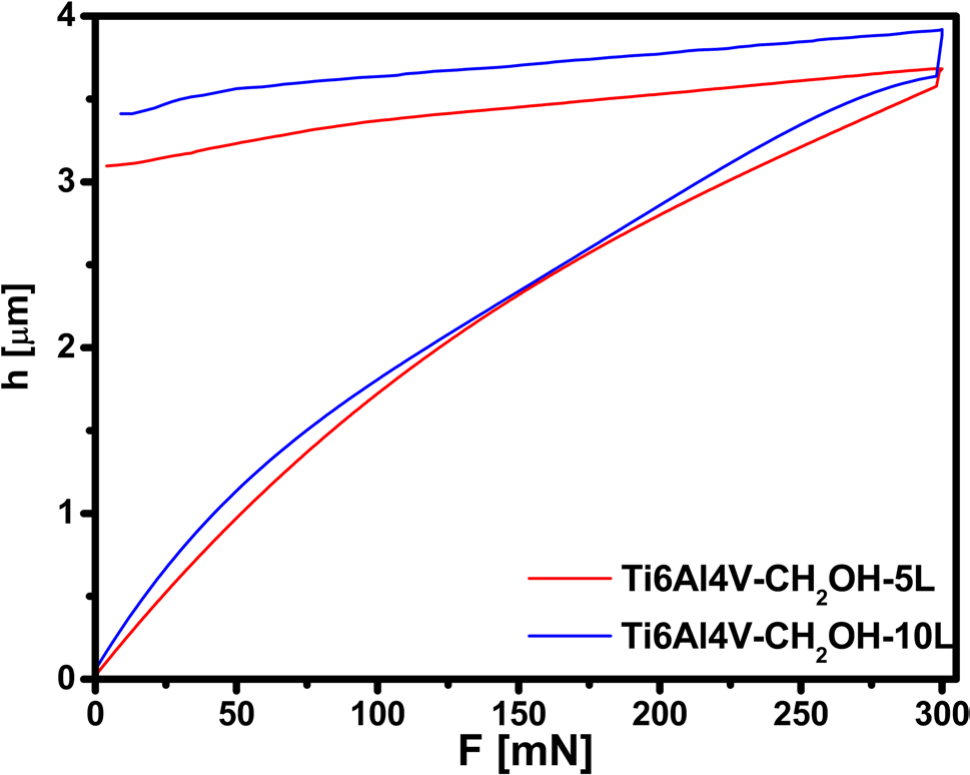
Nanoindentation curve for Ti6Al4V–CH_2_OH–5L and Ti6Al4V–CH–OH–10L.

Load-unload curves for polyurethane layers as a function of the indentation depth are shown in Fig. 6. These results show slight differences between the mechanical properties of both layers^35^. The maximum indentation depth achieved when measuring the mechanical properties is an important parameter that indicates changes in the surface layer^36^. The maximum indentation depth was 3.69 µm for Ti6Al4V–CH_2_OH–5L while 3.92 µm for Ti6Al4V–CH_2_OH–10L. It is seen that only a small deal of the peak-load displacement in Fig. 6 is elastically recovered on unloading. The share of plastic deformation was 82.94% for Ti6Al4V–CH_2_OH–5L while 84.73% for Ti6Al4V–CH_2_OH–10L. On the other hand, elastic recovery for the uncoated Ti6Al4V substrates only 17%. These observations imply that hardness and elastic modulus of the polyurethane coating is lower than those of the Ti6Al4V substrate and indirectly indicate the formation of a thicker polyurethane layer on Ti6Al4V–CH2OH–10L, which has already been confirmed by previous techniques^37^.

**Figure 7:**
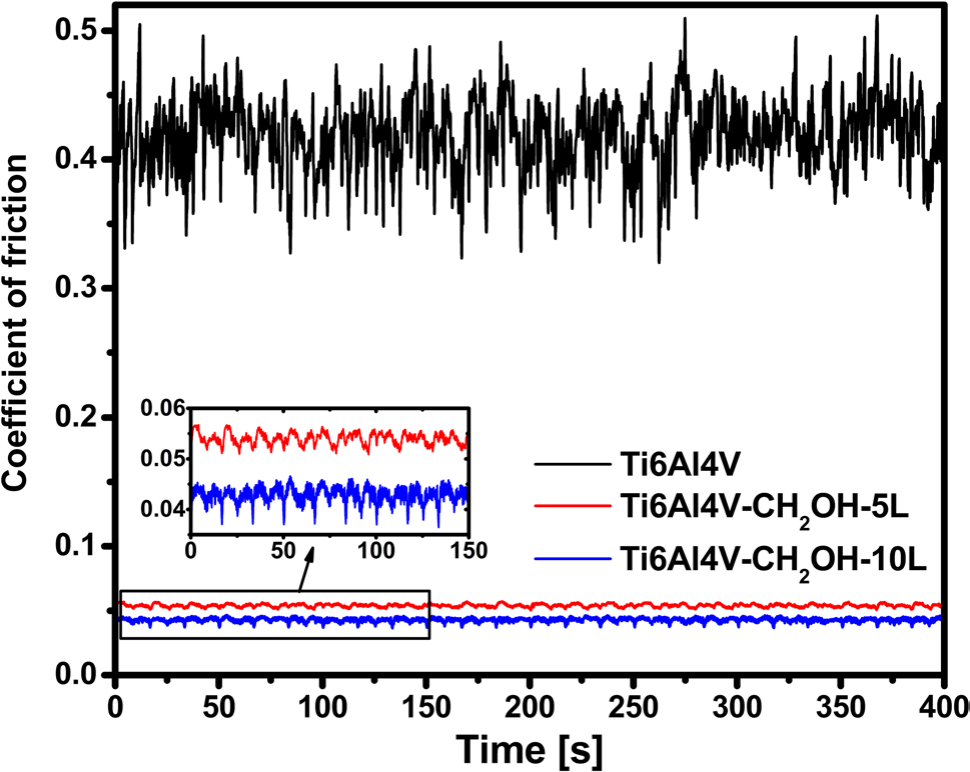
Coefficient of friction for Ti6Al4V–CH_2_OH–5L and Ti6Al4V–CH_2_OH–10L.

The most important goal of the presented research was to improve the abrasive resistance of the titanium alloy. Improving these properties is tantamount to lowering the value of coefficient of friction. Tribology test results obtained for the alloys with a polyurethane layer are presented in the Fig. 7. The influence of the number of polyurethane layers on the abrasion resistance is visible. The alloy with 10 polyurethane layers has a lower coefficient, which indicates its lower susceptibility to abrasion. The results after each cycle are stable and look similar, which proves the stability of the layers during the tribological test.

Compared to unmodified titanium, the coefficient of friction for materials with polymer layers decrease almost 10 times (Table 2). So far, such a low coefficient of friction has not been reported in the literature for modified Ti6Al4V as evidenced by the results presented in Table 2. Low values were obtained, for example, for thermal oxidized Ti6Al4V, but the tests were carried out in lubricant, which indicates that in dry conditions this value could be higher. Despite the research carried out in a lubricant, the layer presented in this work still has over 4 times better properties. The comparison of the obtained results with the literature results confirms the high application potential of the obtained layers as protective layers in endoprosthesis.

**Table 2:** Comparison of the obtained results with the literature data.

	Coefficient of friction	References
Ti6Al4V	0.417 ± 0.031	This work
Ti6Al4V–CH_2_OH–5L	0.054 ± 0.001	This work
Ti6Al4V-CH_2_OH–10L	0.043 ± 0.002	This work
Ti6Al4V	0.5 ± 0.1	^38^
Thermal oxidized Ti6Al4V	0.2	^39^
Si3N4/PEEK 708 coating on Ti6Al4V	0.26	^40^
TiN/PEEK708 coating on Ti6Al4V	0.30	^41^
SiC coating on Ti–6Al–4V	0.39	^42^
Plasma nitrided Ti6Al4V	0.23	^43^

## Conclusions

In this work, we proved the effectiveness of the alloy surface modification with diazonium salts and the possibility of attachment of polyurethane layers to modified alloy. It has been shown that 4-hydroxymethyl benzenediazonium salt is the most effective modifier and the resulting aryl layer is evenly distributed as confirmed by Raman mapping. It has also been found that it is possible to produce polyurethane layers of different thickness. For both five and ten layers, these layers are evenly distributed over the entire surface of the alloy as confirmed by Raman mapping. The effectiveness of attaching subsequent layers of polyurethane was also confirmed by nanoindentation. The most important goal of the presented research was to improve the abrasion resistance of titanium alloy. Coefficient of friction was lower for the titanium alloy with 10 layers of polymer than titanium alloy with 5 layers. In addition, it is about 10 times lower compared to unmodified titanium alloy. The results obtained in this study are the first step to obtain endoprostheses with very high abrasion resistance.
